# The Lone Star Medical Association

**Published:** 1888-05

**Authors:** 


SULPHATE OF SPARTRINE.—This new alkaloid of broom is forging to the front as a substitute for digitalis. Germain See found that it possessed a stimulating action upon the contractions of the heart, slowing its action; and that it increased the arterial tension, and also increased the flow of urine." It seems that spartrine acts very much like digitalis, except that its effects are more transient. Voight claims superior calmative action for the sulphate of spartrine and recommends it in valvular disease, in pericarditis and finally as an adjuvant in combination with digitalis. The dose is about one-third of a grain three times a day. It is soluble in water, and tastes bitter.

